# Association between insulin resistance and cardiac remodeling in HER2-positive breast cancer patients: a real-world study

**DOI:** 10.1186/s12885-023-11102-y

**Published:** 2023-07-03

**Authors:** Yunjing Shi, Zeping Qiu, Jing Yu, Zhuojin Li, Sha Hua, Yanjia Chen, Xiaosong Chen, Kunwei Shen, Wei Jin

**Affiliations:** 1 Department of Cardiovascular Medicine, Ruijin Hospital, Shanghai Jiao Tong University School of Medicine, 197 Ruijin 2nd Road, Shanghai, 200025 P. R. China; 2 Institute of Cardiovascular Diseases, Shanghai Jiao Tong University School of Medicine, 197 Ruijin 2nd Road, Shanghai, 200025 P. R. China; 3 Department of General Surgery, Comprehensive Breast Health Center, Ruijin Hospital, Shanghai Jiao Tong University School of Medicine, Shanghai, 200025 P.R. China; 4 Heart Failure Center, Ruijin Hospital Lu Wan Branch, Shanghai Jiao Tong University School of Medicine, 149 S. Chongqing Road, Shanghai, 200020 P. R. China

**Keywords:** Breast cancer, Insulin resistance, Asymptomatic cancer therapy-related cardiac dysfunction, Left atrial adverse remodeling

## Abstract

**Background:**

Insulin resistance is an overlapping risk factor for both heart and breast cancer, while its interaction with cardiotoxicity in breast cancer (BC) patients is not clear. This study investigated the impact of insulin resistance on cardiac remodeling in patients with human epidermal growth factor receptor 2 (HER2)-positive BC during and after trastuzumab therapy in real-world clinical practice.

**Methods:**

HER2-positive BC patients who received trastuzumab treatment between December 2012 and December 2017 were reviewed and 441 patients with baseline metabolic indices and serial echocardiographic measurements (baseline, 6, 12, and 18 months) after trastuzumab therapy initiation were included. Repeated measurement analysis of variance was used to evaluate temporal trends in multiparameter echocardiography. Linear mixed model was applied to further evaluate the role of insulin resistance in forementioned changes. Correlation of homeostasis model assessment-estimated insulin resistance (HOMA-IR) and triglyceride-glucose index (TyG) levels to changes in echocardiography parameters was explored.

**Results:**

Of 441 patients (mean age 54 ± 10 [SD] years), 61.8% received anthracycline-based chemotherapy, 33.5% received left-sided radiotherapy, 46% received endocrine therapy. No symptomatic cardiac dysfunction was observed over the therapy course. A total of 19 (4.3%) participants experienced asymptomatic cancer therapy-related cardiac dysfunction (CTRCD), and the peak onset time was 12 months after the initiation of trastuzumab. Albeit relatively low CTRCD incidence, cardiac geometry remodeling, especially left atrial (LA) dilation over therapy was notable and was more severe in high HOMA-IR and TyG level groups (*P* < 0.01). Noteworthy, a partial reversibility of cardiac remodeling was observed with treatment cessation. Additionally, HOMA-IR level positively correlated to changes in LA diameter from baseline to 12 months (*r* = 0.178, *P* = 0.003). No significant association (all *P* > 0.10) was detected between HOMA-IR or TyG level and dynamic left ventricular parameter evaluation. Multivariate linear regression analysis demonstrated that higher HOMA-IR level was an independent determinant for LA enlargement in BC patients during anti-HER2 targeted therapy course after adjusting for confounding risk factors (*P* = 0.006).

**Conclusion:**

Insulin resistance was associated with left atrial adverse remodeling (LAAR) in HER2-positive BC patients that received standard trastuzumab therapy, indicating that insulin resistance could be a supplementation to baseline cardiovascular risk stratification proforma for HER2-targeted antitumor therapies.

**Supplementary Information:**

The online version contains supplementary material available at 10.1186/s12885-023-11102-y.

## Clinical perspective

### What is New?

- HFA-ICOS elaborated several risk factors for HER2-targeted cancer therapies, while scarce studies explored novel biomarker for subclinical cardiotoxicity in cardio-oncology.
- We presented a real-world study where insulin resistance, an overlapping risk factor of cardiac remodeling and breast cancer mortality, was associated with left atrial dilation in HER2-positive breast cancer patients receiving standard trastuzumab therapy.

### What are the clinical implications?

- We first demonstrated the causal relation between the prediabetic state and cardiac remodeling in HER2-positive breast cancer patients and supposed insulin resistance as a novel risk factor for subclinical cardiotoxicity of trastuzumab treatment.
- This article provided novel insights into the crosstalk between metabolism, oncology and cardiology, setting the way forward for the cardio-oncology towards multidisciplinary cooperation.

## Introduction

Breast cancer (BC) is the most common malignancy in women all over the world, among which human epidermal growth factor receptor 2-positive (HER2+) BC accounts for 20-30% patients [1]. Advancement in HER2-targeted agents, especially the first monoclonal antibody trastuzumab has shifted HER2-positive BC from an aggressive disease to one with a relatively favorable outlook [2]. Nonetheless, this treatment benefit comes with inevitable cardiac sequelae, which mainly exhibits as left ventricular ejection fraction (LVEF) decline, or even congestive heart failure [3–5]. With the survival prolongation, cardiovascular disease (CVD) was reported to be the prevalent cause of non-relapse-related mortality in HER2-positive BC patients [6, 7].

The Heart Failure Association (HFA) of the European Society of Cardiology together with the International Cardio-Oncology Society (ICOS) proposed charts for baseline cardiovascular (CV) risk stratification of several anticancer therapies [8]. According to the HFA-ICOS score, risk factors for anti-HER2 therapies include older age, pre-existing CVD, previous or concurrent anthracycline use, prior radiotherapy to left chest or mediastinum, obesity, hypertension and diabetes mellitus (DM)[9]. Among these risk factors, centripetal obesity, hypertension, hyperglycemia, hyperinsulinemia are all associated with metabolic syndrome (MeS)[10], which could predispose individuals to both CVD and cancer [7, 11, 12].

Insulin resistance (IR), defined as the uncoordinated glucose-lowering response for target tissues at a normal plasma insulin level, plays a key role in the etiology and pathogenesis of MeS [13, 14]. Homeostasis model assessment-estimated insulin resistance (HOMA-IR) and triglyceride-glucose index (TyG) are two well recognized indictors of insulin resistance [15, 16]. The detrimental effect of IR on CVD and cancer has been illustrated. Mounting evidence revealed that IR mediated the development of atherosclerosis, myocardial infarction and heart failure (HF) to some extent [15, 17, 18]. For instance, a study from the University of Oxford which enrolled 4,344 UKPDS participants discovered that a doubled HOMA2-IR value in newly diagnosed T2DM patients was associated with a 5% greater risk of HF death and a 14% greater risk of HF onset over a median follow-up of 16.4 years [17]. Apart from overt HF, the predictive value of IR for subclinical cardiac adverse remodeling has also been proposed [19–21]. A prospective study evaluated multilayer global longitudinal strain (MGLS) of matched patients with or without IR and concluded that patients with higher HOMA-IR values had significantly lower strain values [22].

In terms of oncology, IR was reported to be associated with higher BC incidence and all-cause mortality [23–25]. In prior studies, severer IR was associated with 34% higher BC incidence and 78% higher all-cause mortality after a median of 19.8 years follow-up of 1328 postmenopausal women [24]. Interestingly, a recent study found that breast cancer would in turn induce insulin resistance via cancer-cell-secreted extracellular vesicles [26]. Additionally, IR played a role in the racial disparities in BC prognosis. Compared to white women, black women with more invasive BC tended to have higher HOMA-IR levels, and IR partly mediated the association between race and worse prognosis [25, 27].

As a hazard for both heart and breast cancer, the impact of IR on cardiotoxicity in HER2-positive BC patients has not been fully explored. Here, we investigated the association between insulin resistance and cardiac remodeling in HER2-positive BC survivors with serial echocardiography measurements during and after trastuzumab therapy in real-world clinical practice.

## Materials and methods

### Study population

**Fig. 1:**
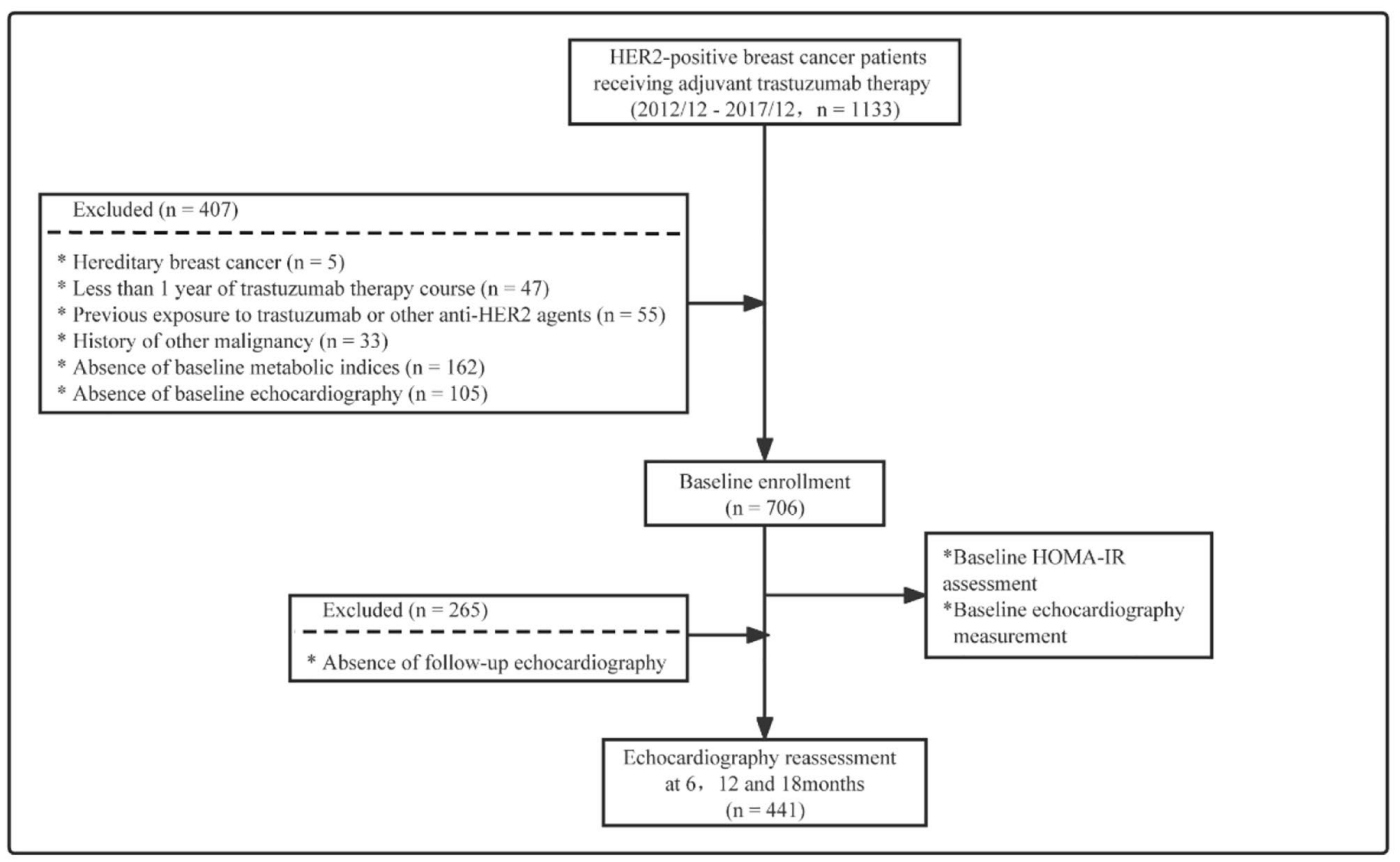
Flow chart of patient enrollment

BC patients who received surgery at General Surgery Department, Comprehensive Breast Health Center, Ruijin Hospital, Shanghai Jiaotong University School of Medicine between December 2012 and December 2017 were retrospectively reviewed. A total of 1133 patients were HER2-positive and received adjuvant trastuzumab therapy (TT). All participants were East Asian. Trastuzumab was intravenously administered with an initial dose of 8 mg/kg followed by a maintenance dose of 6 mg/kg every 3 weeks for 12 months. The main exclusion criteria included: (1) hereditary breast cancer, (2) less than 1 year of TT course, (3) previous exposure to trastuzumab or other anti-HER2 agents, (4) history of other malignancy, (5) absence of baseline metabolic indices or serial echocardiography measurements (baseline, 6, 12, and 18 months). Eventually, 441 participants were enrolled for final analysis (Fig. 1). Asymptomatic cancer therapy-related cardiac dysfunction (CTRCD) was defined as a decrease in LVEF by ≥ 10% below 50% or ≥ 15% from baseline. This study was reviewed and approved by the independent Ethical Committees of Ruijin Hospital, Shanghai Jiao Tong University School of Medicine and written informed consent was obtained from all participants.

### Clinical, pathological and biochemical assessments

Baseline characteristics including demographics, comorbidities, menstrual state, tumor staging, planned surgery, chemotherapy, radiotherapy, endocrine therapy, metabolic indices were retrieved from Shanghai Jiaotong University Breast Cancer Database (SJTUBCDB). The adjuvant therapy scheme for each patient was decided by a multidisciplinary team consisting of breast surgeons, medical oncologists, radiation oncologists, pathologists and specialized breast nurses.

Histopathological evaluation of tumor histological subtype, grade and lymphovascular invasion was conducted by professional pathologists at the Department of Pathology of Ruijin Hospital in accordance with the World Health Organization classification [28]. Estrogen receptor positive (ER+) and progesterone receptor positive (PR+) tumors were defined as tumors with nuclear staining in ≥ 1% of tumor cells. Ki‑67 index was characterized as the proportion of nuclear-stain positive cells to the total number of cells in ≥ 1,000 invasive tumor cells. HER2 positivity was defined as immunohistochemistry score of 3 or fluorescence in situ hybridization positive.

The blood samples were collected in a quiet, air-conditioned room after overnight fasting and after at least 20 min supine rest. Metabolic indices including fasting plasma glucose (FPG), insulin (FINS), total cholesterol (TC), low-density lipoprotein-cholesterol (LDL-C), high-density lipoprotein cholesterol (HDLC), triglycerides (TG) were assessed (HITACHI 912 Analyzer, Roche Diagnostics, Germany). HOMA-IR was calculated according to the formula: HOMA-IR = FINS (µU/L) × FPG (mmol/L) / 22.5. TyG was calculated using the formula: TyG = LN [TG (mg/dl) × FPG (mg/dl)] / 2. Body mass index (BMI) was calculated using the formula of weight/height^2^ (kilograms per square meter). Body surface area (BSA) was calculated using the formula: BSA = 0.0061 × height + 0.0128 × weight − 0.1529.

### Echocardiography examinations

Transthoracic echocardiography was performed before and 6, 12, 18 months after the initiation of TT, via a commercially available system (Vivid-I; GE Healthcare, Milwaukee, WI) with a 1.9-3.8-MHz phased-array transducer. Two dimensional (2D), pulsed-Doppler imaging was performed from standard parasternal and apical transducer positions with 2D frame rates of 60 to 100 frames/s. All data were stored digitally, and offline data analysis was performed (EchoPac, version 7; GE Healthcare) at the termination of the study by two cardiologists blinded to the study members.

Left ventricular dimensions and wall thickness including left ventricular end-systolic diameter (LVESD), end-diastolic diameter (LVEDD), interventricular septal wall thickness (IVST) and LV posterior wall thickness (LVPWT), in diastole (d), were made in the parasternal long axis with the M-mode cursor positioned just beyond the mitral leaflet tips, perpendicular to the long axis of the ventricle. Maximal left atrial diameter (LAD) was also observed in the parasternal long axis. Left ventricular end-diastolic volume (LVEDV) and left ventricular end-systolic volume (LVESV) were measured from the apical four chamber view and calculated by Simpson’s biplane method. The LVEF was determined as the difference between LVEDV and LVESV, relative to the LVEDV. All parameters were measured twice, and the average value was recorded. Relative wall thickness (RWT) was determined by the formula: RWT = (IVST + LVPWT) / LVEDD. LV mass was estimated by the formula: LV mass = 0.8 × 1.04 × (LVEDD + IVST + LVPWT)^3^ - LVEDD^3^ + 0.6. LVEDV, LVESV and LV mass were indexed by BSA calculated at each study time point.

### Statistical analysis

Continuous variables were expressed as the mean and standard deviation (SD) if data were normally distributed or the median with interquartile ranges (IQR) if not. Normal distribution was evaluated with Kolmogorov-Smirnov test. Between-group differences (3 groups) were compared by one-way analysis of variance (ANOVA) if data were normally distributed or Kruskal-Wallis test if not, followed by post hoc Bonferroni test. Categorical data were summarized as proportions and frequencies and analyzed by Chi-square or Fisher’s exact test. Repeated measurement analysis of variance was used to evaluate temporal trends in multiparameter echocardiography. Linear mixed model was conducted to evaluate the impact of IR in forementioned changes. Correlation between IR and changes in echocardiography parameters was determined by Spearman’s correlation test. HOMA-IR was defined as a log-transformed continuous variable in linear regression model analysis and an ordinal variable divided by its tertiles with the lowest tertile as reference to evaluate its value. Multivariable linear regression analysis was implemented to interrogate the independent diagnostic value of HOMA-IR for LAAR. Coefficients were presented after adjusting for age, BMI, hypertension, previous CVD and whether or not receiving anthracycline-based chemotherapy, left-sided radiotherapy, endocrine therapy. All statistical analyses were performed with the SPSS 25.0 for Windows (SPSS, Inc., Chicago, IL, USA). A 2-tailed *P* < 0.05 was considered statistically significant. All authors had full access to the data in the study and took responsibility for the integrity of data and accuracy of data analysis.

## Results

### Basic characteristics of the studied population

**Fig. 2:**
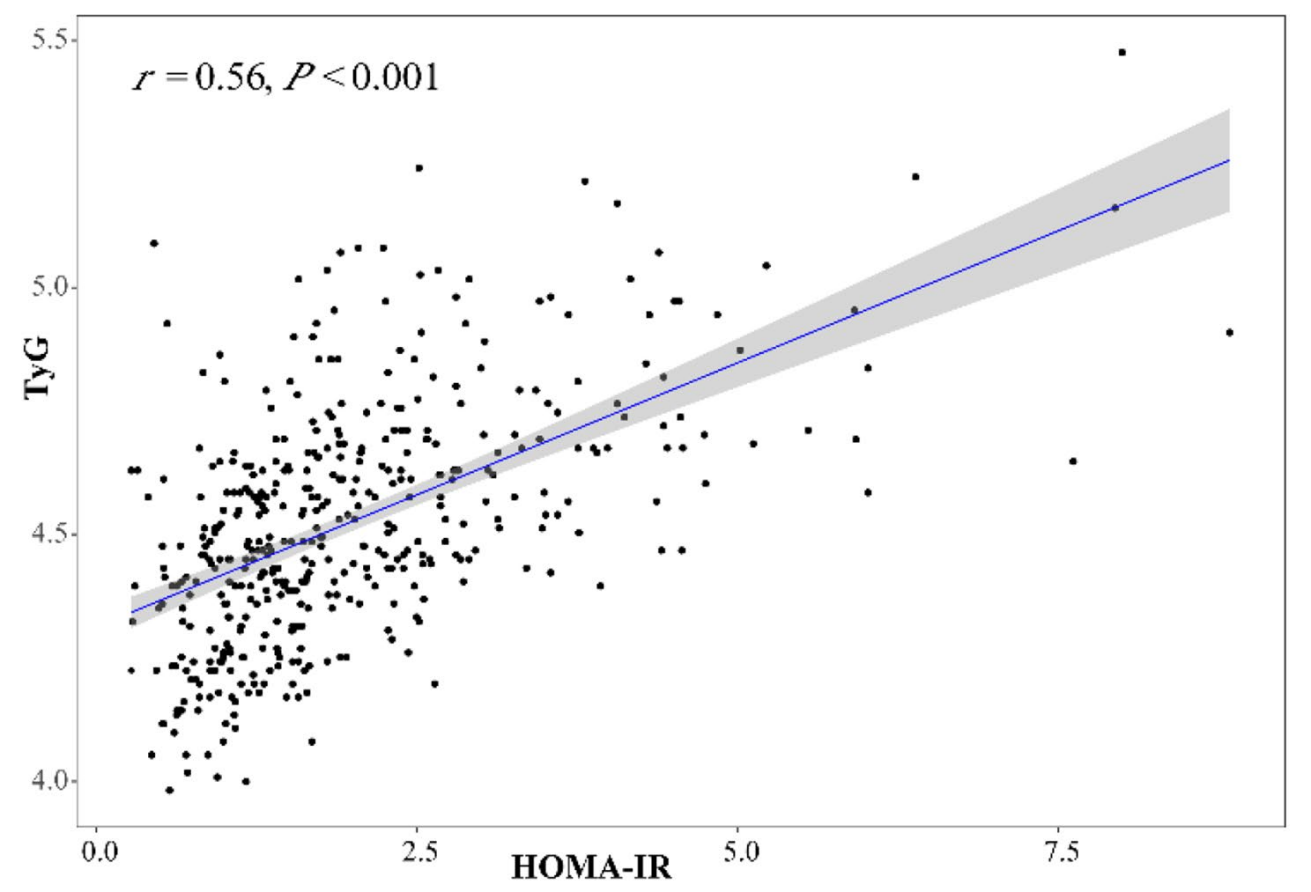
Spearman’s correlation between HOMA-IR and TyG. HOMA-IR homeostasis model assessment-estimated insulin resistance, TyG triglyceride-glucose index

A total of 441 HER2-positive BC patients who received adjuvant TT for 1 year were enrolled in this study. Among them, 61.8% received anthracycline-based chemotherapy, 33.5% received left-sided radiotherapy, 46% received endocrine therapy. The proportion of diabetes and dyslipidemia were 7.7% and 1.8% respectively, while the level of HOMA-IR (1.79 [1.22–2.69]) and TyG (4.57 [4.42–4.75]) was higher than the normal range according to previous reports [29, 30]. The level of HOMA-IR was well correlated with TyG in this study (*r* = 0.560, *P* < 0.001) (Fig. 2). We then classified the study population into low, medium and high HOMA-IR tertiles, which ranged from 0.31 to 1.40, 1.41–2.35 and 2.36–9.51 respectively. It turned out that subjects with higher HOMA-IR level tended to have higher BMI, systolic blood pressure, fasting glucose index, fasting lipid index and more cardiometabolic complications. No between-group differences were present in terms of oncological features and anti-tumor formula (Table 1). As for baseline echocardiographic parameters, no significant differences in LVEF, LV volume, and LV mass were observed between three groups. Nonetheless, as IR had been reported to exert subclinical impact on concentric hypertrophy and left atrial remodeling, ahead of overt cardiac dysfunction, subjects with higher HOMA-IR level had increased LA diameters and thicker wall thickness at baseline (Table S1)[31].

**Table 1:** Baseline characteristics

HOMA-IR tertiles	low	medium	high	*P* - value
n	147	147	147	
Range	0.31–1.40	1.41–2.35	2.36–9.51	
Demographic characteristics				
Age, median (IQR)	54(13)	53(16)	57(12)	0.013
BMI, kg/m^2^, mean (SD)	21.43(3.56)	22.68(3.02)	24.34(3.83)	< 0.001
SBP, mmHg, median (IQR)	122(20)	125(25)	134(27)	< 0.001
DBP, mmHg, median (IQR)	73(13)	74(11)	74(14)	0.713
Menopause, n (%)	85(57.5%)	80(54.4%)	91(61.9%)	0.429
Comorbidity, n (%)				
Hypertension	17(11.6%)	18(12.2%)	46(31.3%)	< 0.001
Diabetes mellitus	8(5.4%)	7(4.8%)	19(12.9%)	0.014
Hyperlipidemia	2(1.4%)	1(0.7%)	5(3.4%)	0.191
Cardiovascular disease^a^	4(2.7%)	5(3.4%)	16(10.9%)	0.004
Renal dysfunction^b^	2(1.4%)	1(0.7%)	1(0.7%)	0.777
Oncological features, n (%)				
TNM stage I-II	107(72.8%)	101(68.7%)	106(72.1%)	0.455
TNM stage III	25(17.0%)	36(24.5%)	32(21.8%)	
Mastectomy	114(77.4%)	108(73.5%)	120(82.9%)	0.416
Breast conserving surgery	28(19.3%)	30(20.4%)	23(15.6%)	
Histology type: IDC	78(53.1%)	86(58.5%)	87(59.2%)	0.655
ER or PR+	66(48.9%)	67(47.2%)	60(42.6%)	0.548
Ki67 ≥ 14	122(83.0%)	126 (85.7%)	127(86.4%)	0.688
Anthracycline-based chemotherapy	90(60.8%)	93(62.4%)	91(61.8%)	0.169
Left-sided radiotherapy	49(33.1%)	54(36.2%)	45(31.0%)	0.635
Endocrine therapy	67(45.6%)	72(49.0%)	66(44.9%)	0.476
Laboratory values, mean (SD)				
Fasting glucose, mmol/L	4.90(0.52)	5.09(0.62)	5.65(1.32)	< 0.001
Fasting insulin, µU/mL	4.80(1.71)	7.73(1.49)	12.58(4.99)	< 0.001
Triglyceride, mmol/L	0.91(0.52)	1.15(0.62)	1.43(0.82)	< 0.001
Total cholesterol, mmol/L	4.75(1.39)	5.03(1.14)	4.89(1.37)	0.049
HDL cholesterol, mmol/L	1.50(0.45)	1.40(0.43)	1.26(0.36)	< 0.001
LDL cholesterol, mmol/L	2.81(1.18)	3.08(1.00)	3.04(0.96)	0.169

^a^Cardiovascular disease referred to arrhythmia, coronary heart disease and myositis

^b^Renal dysfunction is defined as glomerular filtration rate (eGFR) < 60 ml /min/1.73m^2^

BMI body mass index, SBP systolic blood pressure, DBP diastolic blood pressure, IDC invasive ductal carcinoma, ER estrogen receptor positive, PR progesterone receptor positive, HDL high-density lipoprotein, LDL low-density lipoprotein, IQR interquartile range, SD standard deviation.

### Dynamic changes in echocardiographic geometric and functional properties over adjuvant trastuzumab therapy

Transthoracic echocardiography examinations before and 6,12,18 months after the initiation of 1-year course of TT were conducted according to the position statement [32]. At baseline, no one was concomitant with HF or LV systolic dysfunction. Over the therapy course, 5 patients experienced a decline of LVEF ＞15% but still within the normal range at 6 months. The number added up to 16 at 12 months, and 4 of whom had a drop of LVEF below 50%. However, half of the patients partly recovered from CTRCD after the TT termination and only 2 patients retained the LVEF under 50%. Interestingly, the baseline HOMA-IR levels of aforementioned patients suffering from CTRCD were found to be significantly higher than those event-free patients, whenever the event occurred (Table S2).

**Fig. 3:**
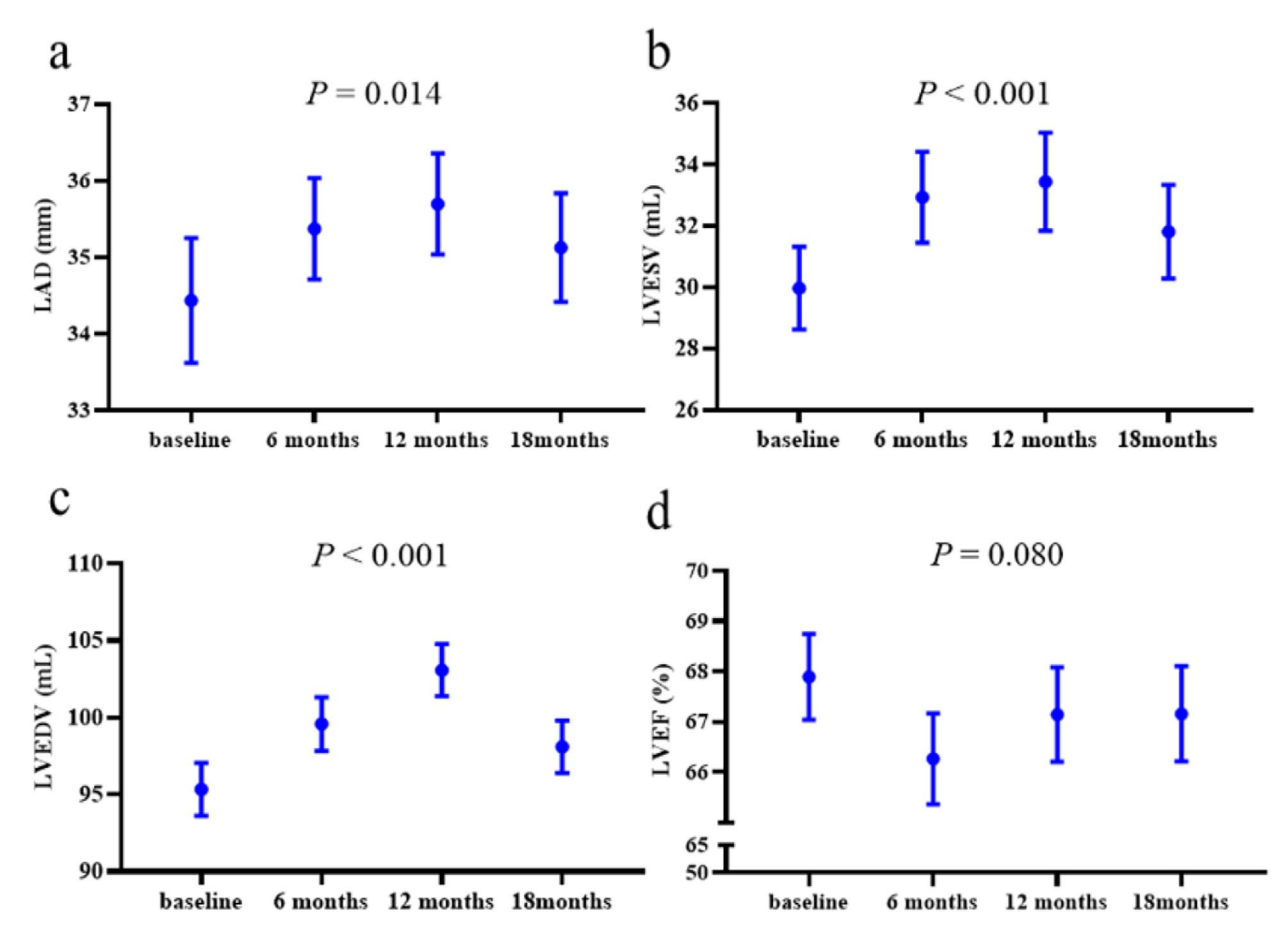
Temporal trends in echocardiography indices before, during and after trastuzumab therapy. LAD left atrial diameter, LVESV left ventricular end-systolic volume, LVEDV left ventricular end-diastolic volume, LVEF left ventricular ejection fraction. *P* values in the graph titles refer to overall comparisons across 4 time points via repeated measurement analysis of variance. Vertical bars indicate 95% confidence interval (CI).

**Fig. 4:**
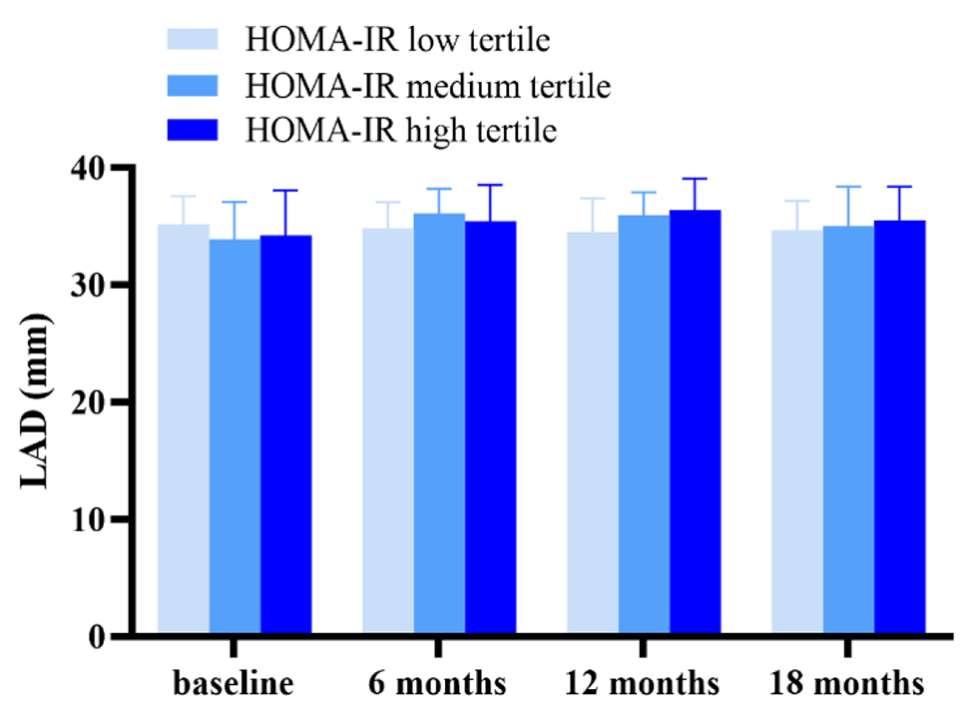
Temporal trends in LAD before, during and after trastuzumab therapy among HOMA-IR tertiles. HOMA-IR homeostasis model assessment-estimated insulin resistance, LAD left atrial diameter

Repeated measurement analysis of variance was used to evaluate longitudinal multiparameter changes of echocardiography over the course of TT. Albeit relatively low CTRCD incidence, there was a deterioration of cardiac geometry structure and function manifested as LA enlargement (*P* = 0.014), LV dilation (LVESV, LVEDV, both *P* < 0.001) and LVEF decline (*P* = 0.080) from baseline to 12 months, while the situation was partly reversed 6 months after the cessation of TT (Fig. 3). Additionally, when grouped by HOMA-IR tertiles, only the temporal changes of LA parameter were significantly different between groups, indicating that IR degree exerted impact on LA remodeling in BC patients over the TT (*P*_*int*_ = 0.007, Fig. 4).

**Fig. 5:**
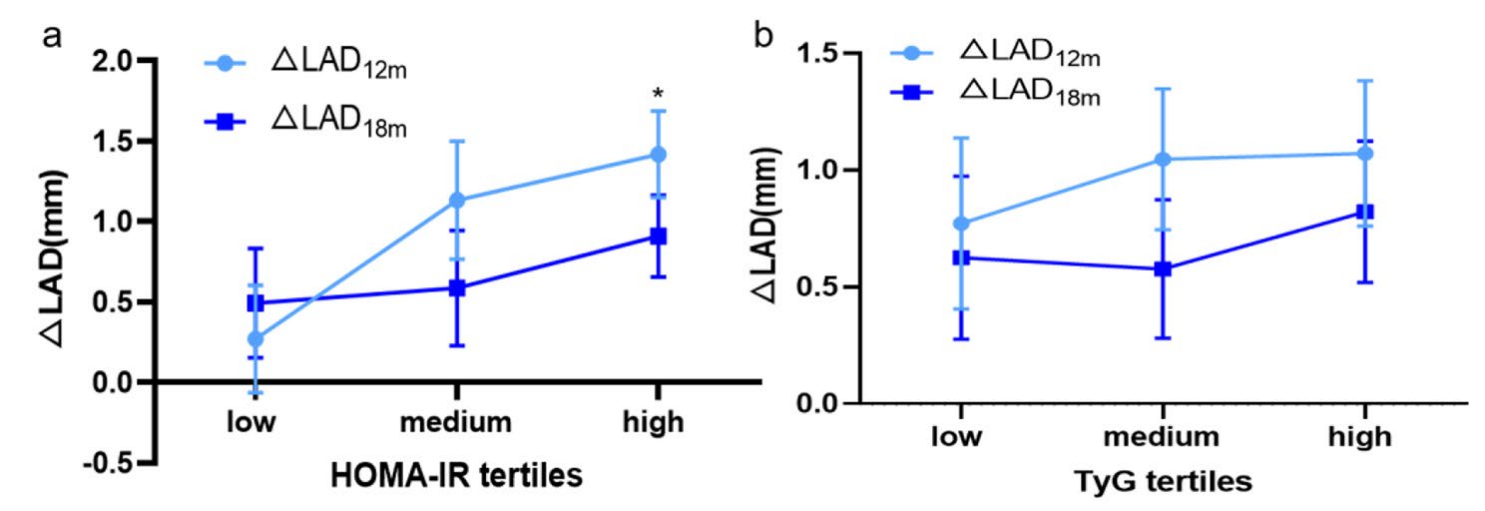
Distribution of changes in LAD at 12 months and 18months after the trastuzumab therapy compared to baseline among HOMA-IR (a) and TyG (b) tertiles. HOMA-IR homeostasis model assessment-estimated insulin resistance, TyG triglyceride-glucose index, LAD left atrial diameter, Data are expressed as mean ± 95% confidence interval. One-way ANOVA with Bonferroni multiple comparisons test was used. *P < 0.05

To further assess the impact of IR on cardiac remodeling, follow-up echocardiography parameters were compared in subjects stratified by HOMA-IR and TyG levels (Table 2, S3). No significant differences were discovered between groups in terms of LVEF that tended to be the main metric of CTRCD, so as other LV parameters. Nonetheless, we found LA diameter increased with increasing tertiles of HOMA-IR and TyG at follow-up time points, which raised our interest. Hence, changes of LA diameters from baseline between different levels of HOMA-IR and TyG tertiles were explored. Interestingly, there was an upward inclination in LA dilation at 12 months and 18 months compared to baseline with increasing tertiles of HOMA-IR (ΔLAD_12m_, *P* < 0.05). Likewise, a similar trend was observed between different groups of TyG at 12 months (Fig. 5). Above all, we preliminary supposed that LA dilation over therapy was significant and was more severe in high IR level groups.

**Table 2:** Follow-up echocardiography parameters grouped by HOMA-IR tertiles over the trastuzumab therapy course

Tertiles		HOMAIR low	HOMA-IR medium	HOMA-IR high	*P* - value
LAD					
0	33.75(3.54)		34.39(3.27)	35.28(3.58)	0.003
6 m	34.32(3.31)		35.46(3.41)	35.69(3.30)	0.035
12 m	34.31(3.56)		35.32(3.15)	35.98(3.38)	0.002
18 m	34.26(3.62)		34.85(3.18)	36.15(3.08)	< 0.001
LVEF					
0	68.15(3.71)		67.91(3.84)	68.25(3.88)	0.787
6 m	66.29(4.45)		66.75(3.85)	67.10(3.94)	0.504
12 m	66.00(4.84)		66.07(4.25)	65.63(5.42)	0.780
18 m	65.87(4.22)		65.98(3.85)	66.33(4.54)	0.716
LVMi					
0	74.90(13.70)		76.53(12.11)	77.21(13.49)	0.396
6 m	78.39(14.29)		79.46(13.03)	78.65(12.42)	0.887
12 m	78.80(10.77)		79.81(12.80)	80.66(12.44)	0.545
18 m	77.58(13.55)		77.07(11.84)	79.24(13.34)	0.457
RWT					
0	0.371(0.034)		0.370(0.036)	0.392(0.047)	< 0.001
6 m	0.368(0.039)		0.373(0.040)	0.380(0.042)	0.231
12 m	0.362(0.031)		0.368(0.035)	0.376(0.038)	0.016
18 m	0.362(0.035)		0.368(0.037)	0.374(0.038)	0.070

Values are given as mean (SD)

HOMA-IR homeostasis model assessment-estimated insulin resistance, LAD left atrial diameter, LVEF left ventricular ejection fraction, LVMi left ventricular mass indexed to body surface area, RWT relative wall thickness.

### The association between insulin resistance and LA dilation in breast cancer patients during anti-HER2 targeted therapy course

**Fig. 6:**
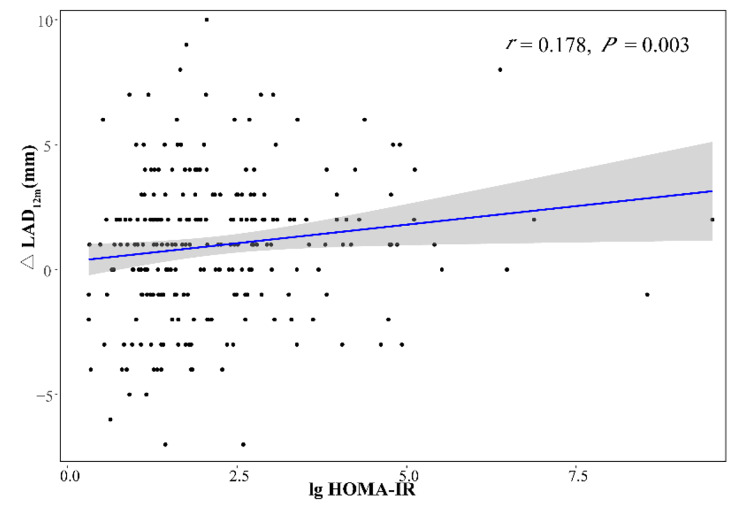
Spearman’s correlation between Log-transformed HOMA-IR and changes in LA dimension from baseline to 12 months. HOMA-IR homeostasis model assessment-estimated insulin resistance, LAD left atrial diameter

As HER2-positive BC patients experienced the severest cardiac remodeling at 12 months after the initiation of TT, correlation between HOMA-IR and echocardiographic parameters changes at 12 months was further investigated. And it turned out that Log-transformed HOMA-IR level positively correlated to changes in LA diameter from baseline to 12 months (*r* = 0.178, *P* = 0.003) (Fig. 6). No significant association (all *P* > 0.10) was detected between HOMA-IR or TyG level and dynamic LV diameter and volume parameter evaluation (Table S4).

Finally, multivariate linear regression was performed to interrogate the association between IR and LA dilation (Table 3). In model 1, after adjusting for confounding clinical variables including age, BMI, hypertension and prior cardiovascular disease, HOMA-IR (as a log-transformed continuous variable) was independently associated with changes in ΔLAD_12m_ [*β* (95%CI): 2.19 (0.64–3.75), *P* = 0.006]. Compared with the low tertile, high tertiles of HOMA-IR corresponded to 0.87-mm and 1.13-mm increases in LAD. In model 2, we further included whether or not receiving anthracycline-based chemotherapy, left-sided radiotherapy, endocrine therapy to rule out the impact of different treatment regimens [*β* (95%CI): 1.69 (0.13–3.25), *P* = 0.034]. In summary, higher HOMA-IR level was an independent determinant for LA enlargement. In a word, insulin resistance played a role in suboptimal cardiovascular health in BC patients during TT course.

**Table 3:** Multivariable linear regression models for HOMA-IR as a risk factor for LA remodeling

	Model 1			Model 2		
Variables	Coefficient (95% CI)	Sβ	*P* – value	Coefficient (95% CI)	Sβ	*P* - value
lg HOMA-IR per SD	2.19(0.64–3.75)	0.196	0.006	1.69(0.13–3.25)	0.151	0.034
First tertile	1 (Reference)		…	1 (Reference)		…
Second tertile	0.87(-0.06-1.80)	0.140	0.065	0.81(-0.11-1.74)	0.130	0.086
Third tertile	1.13(0.19–2.07)	0.190	0.019	0.95(0.00-1.90)	0.159	0.050

Model 1, adjusted for age, body mass index, hypertension, cardiovascular disease

Model 2, adjusted for age, body mass index, whether or not receiving anthracycline-based chemotherapy, left-sided radiotherapy, endocrine therapy

HOMA-IR homeostatic model assessment-insulin resistance, CI confidence interval, SD standard deviation

## Discussion

The major finding of the current study was that relatively low CTRCD incidence but significant cardiac remodeling, especially LA enlargement, existed in HER2-positive BC patients receiving standard anti-tumor therapy. And insulin resistance was an independent determinant for LA enlargement that reflected subclinical cardiotoxicity.

Monitoring of cardiac function is of critical importance for CTRCD surveillance and prevention in BC patients. Echocardiography is recommended as the first-line modality for the assessment of cardiac function in patients with cancer [33]. Serial measurement of LVEF is the most widely applied approach, while it is insensitive for detecting early cardiac dysfunction in case of contractility reserve. As the early detection and prompt intervention of minor cardiac damage are increasingly emphasized in recent years, cardio-oncology appeals for more sensitive indicators that assess diastolic function or myocardial lesions [5, 34]. Among these evaluation metrics, LAAR has been widely reported to be an early sign of asymptomatic cardiac dysfunction despite preserved systolic and diastolic function [35, 36]. In a recent large-scale community-based study which enrolled 2221 healthy participants, multiple echocardiographic parameters of LA were found to be related to HF development after a median follow-up of 15.8 years. Among which the minimum LA volumes indexed to BSA (LAVimin) was the strongest predictor of incident HF [37].

In our study in the non-clinical trial setting, the rate of asymptomatic CTRCD was low probably due to overall low HFA-ICOS baseline CV risk scores. Nonetheless, LAAR trend observed over TT course should not be overlooked for that it might be the herald of late cardiac complications. In line with our study, Flora et al. observed that HER2-positive BC patients suffered from significant decline in LA ejection fraction and increase in LA minimum volume during and after TT via a small prospective 2-center longitudinal study [34]. Lisi et al. discovered that alteration of LA strain (LAS) and LA stiffness index (LASI) preceded that of LV parameters, LV global longitudinal strain (GLS) included, in identifying asymptomatic TT related cardiac dysfunction [38]. Moreover, since trastuzumab-induced cardiotoxicity was considered to be reversible, TT related LAAR was also partly reversed 6 months after the cessation of the course in our cohort.

HFA-ICOS had elaborated several risk factors for HER2-targeted cancer therapies, while scarce studies explored novel biomarkers for subclinical cardiotoxicity in cardio-oncology. IR was reported to be associated with adverse cardiac remodeling and poor prognosis of BC as well. As reported by prior studies, higher level of HOMA-IR was accompanied by lower LAS and higher LASI in general population [39, 40]. IR also increased the susceptibility to incident atrial fibrillation (AF) and AF recurrence after ablation partly due to the atrial electrical remodeling [41, 42]. In this study, we first found that higher IR degree was responsible for more significant LAAR but not LV structural and functional deterioration during and after TT. With increasing tertiles of HOMA-IR or TyG, an upward inclination in LA dilation persisted till 6 months after therapy cessation though the changes of LA diameters from baseline between different levels of HOMA-IR was only statistically significant at 12 months (ΔLAD_12m_, *P* < 0.05). After adjusting for confounding clinical variables, including well-recognized risk factors for HER2-targeted cancer therapies proposed by HFA-ICOS, IR was independently associated with LA enlargement in HER2-positive BC patients. Furthermore, 61.8% received anthracycline-based chemotherapy and 33.6% underwent left-sided radiotherapy in our cohort. Anthracycline application moderately modified the association of HOMA-IR with changes in LAD at 12months, suggesting that the combination of anthracycline and trastuzumab exacerbated the risk of LAAR as well. Nonetheless, forementioned effect was not present in patients coincided with left-sided radiotherapy.

The underline mechanism may be the sophisticated interaction between IR, myocardial dysfunction, BC biology and HER2 signaling. First, IR related with numerous adverse effects in the heart including perturbation in myocardial energy metabolism, mitochondrial dysfunction, endoplasmic reticulum stress, calcium homeostasis imbalance and immune response incoordination. These pathological changes fired the susceptibility of myocardial damage in that HER2 inhibition also increased ROS production, induced mitochondrial function deterioration and diminished cardioprotective effects of neuregulin 1[43]. Secondly, insulin receptor, insulin-like growth factor 1 receptor ( IGF-1R) and HER2 all belong to the tyrosine kinase receptor superfamily, insulin could directly or indirectly activated the IR/IGF-1R/HER2 signaling pathway [44, 45], promoting the growth and metastasis of BC cells, contributing to trastuzumab resistance and trastuzumab-induced cardiotoxicity in HER2-positive BC patients [46, 47]. Thirdly, systemic IR may contribute to myocardial IR, pushing forward the cardiac remodeling in early stage [48]. Myocardial insulin signaling includes two key pathways. The first pathway involving insulin receptor substrate 1/ phosphatidylinositol 3-kinase (IRS-1/PIK3) predominantly elicits metabolic responses, while the second pathway via mitogen-activated protein kinase (MAPK) mainly exerts growth promoting effects, contributing to the resultant myocardial hypertrophy and cardiac fibrosis [49]. IR status could reflect an imbalance between the metabolic and growth pathways of insulin signaling in the myocardium, with the actions of the MAPK predominating over the increased phosphorylated IRS-1/PI3K pathway [50]. Herein, IR exerted a complicated and profound impact on myocardial function in BC patients receiving anti-HER2 therapy.

The primary prevention of CV toxicity emphasizes lifestyle optimization and early intervention of CV risk factors, namely giving propriety to cardiometabolic health management. In our study, we first demonstrated the causative relation between this prediabetic state and cardiac remodeling in HER2-positive BC patients and supposed IR as another novel risk factor for asymptomatic CTRCD. Moreover, we provided a novel insight into the crosstalk between metabolism, oncology and cardiology, setting the way forward for the cardio-oncology towards multidisciplinary cooperation.

### Study limitations

We appreciate limitations in our study. First, this was a retrospective study based on prospectively collected data. Second, the evaluation of LA and LV remodeling was limited to echocardiography parameters. Cardiac magnetic resonance would provide more information. Further prospective studies are warranted to comprehensively assess the impact of IR on the composite endpoint integrating cardiology and oncology.

## Conclusions

In conclusion, this study revealed that insulin resistance was associated with LA dilation in HER2-positive BC patients receiving 1-year of TT in real-world clinical practice, which could be recognized as a sensitive indicator for asymptomatic CTRCD. We appeal for the cardiometabolic health management in BC patients since appropriate intervention of IR would promote a win-win outcome for both heart and breast cancer.

## Electronic supplementary material

Below is the link to the electronic supplementary material.

Supplementary Material 1

## Data Availability

All data generated or analyzed during this study are included in this published article and its supplementary information files.

## Abbreviations

HER2: human epidermal growth factor receptor 2
HOMA-IR: homeostasis model assessment-estimated insulin resistance
TyG: triglyceride-glucose index
CTRCD: cancer therapy-related cardiac dysfunction
LAAR: left atrial adverse remodeling
BC: breast cancer
HFA: Heart Failure Association
ICOS: International Cardio-Oncology Society
MeS: metabolic syndrome
IR: insulin resistance
MGLS: multilayer global longitudinal strain
LAS: LA strain
LASI: LA stiffness index
GLS: global longitudinal strain
IGF-1R: insulin-like growth factor 1 receptor
IRS-1/PIK3: insulin receptor substrate 1/ phosphatidylinositol 3-kinase
MAPK: mitogen-activated protein kinase
