# Supplementary material for: Association between insulin resistance and cardiac remodeling in HER2-positive breast cancer patients: a real-world study
Source: BMC Cancer. 2023 Jul 3;23:615. doi: 10.1186/s12885-023-11102-y (PMC10316574; doi:10.1186/s12885-023-11102-y)
Supplement: Supplementary file 1 — Supplementary Material 1 [file 12885_2023_11102_MOESM1_ESM.docx]

**Supplement materials**

Table S1 Baseline echocardiography parameters grouped by HOMA-IR tertiles

| HOMA‑IR tertiles | low | medium | high | *P -* value |
| --- | --- | --- | --- | --- |
| LVEF | 68.15(3.71) | 67.91(3.84) | 68.25(3.88) | 0.787 |
| LVESV, mL | 28.81(6.20) | 30.15(6.06) | 29.35(7.07) | 0.296 |
| LVEDV, mL | 91.97(15.62) | 95.83(14.30) | 93.70(16.82) | 0.181 |
| LVEDD, mm | 44.67(3.19) | 45.46(2.89) | 45.02(3.37) | 0.176 |
| LVESD, mm | 27.39(2.42) | 28.01(2.82) | 27.67(2.59) | 0.163 |
| LVMi, g/m^2^ | 74.90(13.70) | 76.53(12.11) | 77.27(13.49) | 0.396 |
| IVST, mm | 8.33(0.836) | 8.45(0.838) | 8.86(0.910) | < 0.001 |
| LVPWT, mm | 8.18(0.780) | 8.32(0.741) | 8.70(0.897) | < 0.001 |
| RWT, mm | 0.3706(0.339) | 0.3700(0.036) | 0.3918(0.047) | < 0.001 |
| LAD, mm | 33.75(3.54) | 34.39(3.27) | 35.28(3.58) | 0.003 |

## Values are given as mean (SD)

HOMA-IR homeostasis model assessment-estimated insulin resistance, LVEF left ventricular ejection fraction, LVESV left ventricular end-systolic volume, LVEDV left ventricular end-diastolic volume, LVEDD left ventricular end-diastolic diameter, LVESD left ventricular end-systolic diameter, LVMi left ventricular mass indexed to body surface area, IVST interventricular septal thickness, LVPWT left ventricular posterior wall thickness, RWT relative wall thickness, LAD left atrial diameter

Table S2 Trastuzumab induced cardiotoxicity incidence over the trastuzumab therapy course

| Study time points | baseline | 6 months | 12 months | 18 months |
| --- | --- | --- | --- | --- |
| Asymptomatic CTRCD, n | / | 5 | 16 | 8 |
| LVEF < 50%, n | 0 | 0 | 4 | 2 |
| Baseline HOMA-IR, mean (SD) | / | 1.82(0.80) | 3.16(1.74) | 2.52(1.22) |

CTRCD, cancer therapy-related cardiac dysfunction, LVEF left ventricular ejection fraction, HOMA-IR homeostasis model assessment-estimated insulin resistance

Table S3 Follow-up echocardiography parameters grouped by TyG tertiles over the trastuzumab therapy course

| Tertiles | TyG low | TyG medium | TyG high | *P* - value |
| --- | --- | --- | --- | --- |
| LAD | | | | |
| 0 | 33.51(3.02) | 34.74(3.83) | 35.15(3.44) | 0.001 |
| 6m | 34.72(3.23) | 35.03(3.49) | 35.69(3.39) | 0.241 |
| 12m | 35.14(3.43) | 34.23(3.56) | 35.69(3.09) | 0.003 |
| 18m | 34.07(3.14) | 35.41(3.60) | 35.90(3.16) | < 0.001 |
| LVEF | | | | |
| 0 | 68.22(4.18) | 67.86(3.96) | 68.23(3.31) | 0.701 |
| 6m | 66.74(4.25) | 66.11(4.15) | 67.32(3.87) | 0.229 |
| 12m | 65.71(4.79) | 65.65(4.59) | 66.34(5.26) | 0.543 |
| 18m | 65.50(4.57) | 65.82(3.93) | 66.91(4.03) | 0.047 |
| LVMi | | | | |
| 0 | 73.54(11.91) | 76.50(12.27) | 78.39(14.46) | 0.018 |
| 6m | 78.63(13.73) | 76.93(10.56) | 81.07(15.08) | 0.189 |
| 12m | 77.91(11.37) | 79.71(12.62) | 81.67(11.80) | 0.098 |
| 18m | 76.44(10.92) | 78.48(13.04) | 79.13(14.64) | 0.310 |
| RWT | | | | |
| 0 | 0.3665(0.034) | 0.3783(0.037) | 0.3874(0.047) | < 0.001 |
| 6m | 0.3697(0.038) | 0.3685(0.039) | 0.3825(0.042) | 0.085 |
| 12m | 0.3641(0.038) | 0.3663(0.037) | 0.3756(0.030) | 0.055 |
| 18m | 0.3628(0.032) | 0.3699(0.039) | 0.3727(0.039) | 0.145 |

Values are given as mean (SD)

TyG triglyceride-glucose index, LAD left atrial diameter, LVEF left ventricular ejection fraction, LVMi left ventricular mass indexed to body surface area, RWT relative wall thickness

Table S4 Spearman’s correlation analysis of Log-transformed HOMA-IR & TyG and ∆ echocardiography parameters from baseline to 12 months

| ∆ Echocardiography parameters | Lg HOMA‑IR | TyG |
| --- | --- | --- |
| Δ LVEF_12m_ | 0.031 | 0.022 |
| Δ LVESV_12m_ | 0.024 | 0.050 |
| Δ LVEDV_12m_ | 0.048 | 0.060 |
| Δ LVESD_12m_ | 0.016 | 0.028 |
| Δ LVEDD_12m_ | 0.060 | 0.044 |
| Δ LVMi_12m_ | -0.007 | -0.019 |
| Δ RWT_12m_ | -0.065 | -0.081 |
| Δ LAD_12m_ | 0.178^**^ | 0.087 |

***P* < 0.01

HOMA-IR homeostasis model assessment-estimated insulin resistance, TyG triglyceride-glucose index, LVEF left ventricular ejection fraction, LVESV left ventricular end-systolic volume, LVEDV left ventricular end-diastolic volume, LVEDD left ventricular end-diastolic diameter, LVESD left ventricular end-systolic diameter, LVMi left ventricular mass indexed to body surface area, RWT relative wall thickness, LAD left atrial diameter
